# Effects of Changes in Frequency of Going Out during the COVID-19 Pandemic on *ikigai* (Sense of Purpose in Life) and Mental Health in Middle-Aged and Older Adults in Japan

**DOI:** 10.1007/s10823-024-09504-x

**Published:** 2024-05-01

**Authors:** Takeshi Watanabe, Kai Tanabe, Akiko Tsukao, Shinya Kuno

**Affiliations:** 1 R&D Center for Smart Wellness City Policies, University of Tsukuba, Tsukuba, Japan; 2 Faculty of Health and Sport Sciences, University of Tsukuba, Tsukuba, Japan; 3 Tsukuba Wellness Research Co., Ltd., Chiba, Japan

**Keywords:** COVID-19, *Ikigai*, Physical activity, Sense of purpose in life, Social activities, Well-being

## Abstract

To clarify whether changes in frequency of going out due to the COVID-19 pandemic affect *ikigai* (sense of purpose in life) and mental health in Japanese middle-aged and older adults. In a questionnaire survey mailed to 16,866 adults aged > 40 years in Japan in September 2020, 7,973 responses were received (response rate, 47.3%) in October 2020. Following exclusions, data from 6,978 individuals (50.6% female, mean age 67.8 ± 12.2 years) were available for analysis. Respondents were categorized based on changes in frequency of going out, reflecting changes in social and/or physical activity, during the pandemic compared with before it: the previously active group went out often before but less often during the pandemic; the remained active group continued going out often; and the inactive group continued not going out often. Whether these changes affected the respondents’ *ikigai* and mental health was investigated. The previously active group had a significantly higher proportion of individuals with decreased *ikigai* during the pandemic than the other groups. Mental health score decreased in all groups during the pandemic, but more so in the previously active group (-3.21), followed by the inactive and then the remained active groups (-1.45 and -1.28, respectively). Previously active individuals showed the greatest decline in *ikigai* and mental health among the three groups. These findings suggest that continuing to engage in appropriate physical and social activities, including going out, while following appropriate infection control measures, even under restrictions, can help people maintain *ikigai* and mental health.

## Introduction

The COVID-19 pandemic, which was declared by the World Health Organization (WHO) on 11 March 2020 (Cucinotta & Vanelli, 2020), has subsided somewhat but has yet to end. On 7 April 2020, the Japanese government declared a State of Emergency under Special Measures for Pandemic Influenza and New Infectious Diseases (Cabinet Public Affairs Office, Cabinet Secretariat, 2020). Although the emergency declaration was subsequently lifted and then reimposed as quasi-states of emergency, measures in Japan were not as severe as those in other countries, where strict lockdowns were frequently implemented. Instead, the Japanese government requested citizens to practice social distancing and voluntarily refrain from going out as part of the measures to help prevent the spread of infection. These measures have changed the way people interact with others. Many people have engaged more in virtual communication, such as via social networking services, and less in outside activities and interactions, including direct conversations with others (Onishi et al., 2021). In addition, it has been reported that people who have close associations with others have been better able to maintain a sense of purpose in life, known as *ikigai* in Japanese, compared with those who have less close associations (Office, Government of Japan, 2009). Accordingly, it would seem plausible that self-imposed restrictions on going out due to COVID-19 may be adversely affecting the closeness of associations with others and *ikigai*.

The concept of *ikigai* is now well-recognized in Japan. The government has focused on increasing *ikigai* among older adults to promote longer and healthier lives (Ministry of Health, Labour & Welfare, 2017). The concept of *ikigai* has been translated variously as “self-actualization”, “meaning of life”, and “purpose in life” (Hasegawa et al., 2003), and the government tends to use it to describe the feeling of being alive and having a sense of purpose in living. Thus, in this study, *ikigai* refers to “a sense of purpose in life”. Having a stronger sense of purpose in life was previously found to be associated with less decline in cognitive function (Boyle et al., 2012), lower risks of cardiovascular disease and mortality, and longer and healthier life expectancy (Alimujiang et al., 2019). Conversely, having a lower sense of purpose in life was found to be associated with worse mental health (Neville et al., 2018). It is therefore important to consider how the COVID-19 pandemic could affect *ikigai*.

When considering the effects of a natural disaster like a pandemic on *ikigai* and mental health, we should keep in mind that one of the worst natural disasters in modern history, the 2011 Great East Japan Earthquake, was found to be associated with a loss of *ikigai* and a decline in mental health (Kusano & Fujita, 2017; Omori, 2019). Moreover, a previous investigation of mental health status during the COVID-19 pandemic in Japan has reported a general decline in mental health (Sato et al., 2020). If the current pandemic is adversely affecting *ikigai*, it would be a worrying social and public health issue. However, to our knowledge, no studies have examined how going out less often during the pandemic has affected *ikigai* and mental health status and whether some people have been affected more affected than others. Therefore, the objective of this study was to investigate the impact of pandemic-related changes in the frequency of going out, reflecting changes in social and physical activity, on *ikigai* and mental health in Japan.

## Methods

### Participants

Participants were 16,866 men and women aged > 40 years and living in mountainous countryside areas in Japan (Yawata, Takaishi, and Mitsuke cities and Misato, Shirako, and Higashikagura towns) who were selected using a stratified random sampling method. They were sent the “Survey on secondary health-related impacts associated with the new COVID-19 pandemic” in September 2020. The survey forms were collected online and through the post in October 2020. In total, 7,973 responses were received (response rate: 47.3%). The inclusion criteria in this study were complete answers for the items on age, sex, frequency of going out, and *ikigai*.

The data were anonymized so that individuals would not be identifiable. This study was reviewed and approved by the Ethics Committee of the Faculty of Human Sciences, University of Tsukuba.

### Measurements

A self-administered questionnaire was used to collect information on demographics and lifestyle habits, including, age, sex, height, weight, employment status, household size, and drinking and smoking habits. The questionnaire included two questions about the COVID-19 pandemic. Participants were asked to evaluate their situation both before the pandemic, specifically between April 2019 and January 2020, and at the time of the survey, using the following questions: “How would you evaluate your situation before the COVID-19 pandemic?” and “How would you evaluate your situation today?” Because it can be difficult to precisely characterize the situation before a pandemic or disaster, questions asking about the months leading up to an event have been used in epidemiological studies of the East Japan Earthquake (Konno et al., 2015) as well as COVID-19 (Feter et al., 2021). Based on that, participants were asked whether they had left the house for more than 30 min. “Going out” was defined as leaving the house for more than 30 min for any reason regardless of the type of activity. This did not, however, include going outside to take out the trash or do gardening or yard work. Although several *ikigai* scales have been reported (Imai et al., 2012; Kondo & Kamada, 2003), for the purpose of the present study, we referred a questionnaire used in a previous survey conducted after the Great East Japan Earthquake (Iwate Prefecture and Council of Social Welfare, 2017) in order to make it as easy as possible for the respondents to relay their current feelings about *ikigai*. Participants were asked to respond to the question, “To what extent do you feel *ikigai* (contentment or enjoyment)?” using a 5-point scale ranging from “1. Extremely” to “5. Not at all,” based on the pre-pandemic and present definitions described above. To assess mental health, we used the mental health question items from the WHO-5 Well-Being Index (Awata et al., 2007). To assess the frequency of going out for more than 30 min per week, before and during the COVID-19 pandemic, we used a 5-point scale: “6 or more days a week”, “4–5 days a week”, “2–3 days a week”, “1 day a week” and “1–3 times a month or less”. Given that other studies concluded that care costs are higher for those who go out 2–3 days a week compared with those who go out almost every day (Hirai et al., 2021), we decided to use going out 2–3 days a week as means to categorize participants by frequency. In a preliminary analysis, we found that individuals who were going out more than 2–3 days a week before the pandemic (i.e., 4–5 days a week or 6 or more days a week), tended to be divided into two groups: those whose frequency of going out decreased significantly and those whose frequency remained the same. On the other hand, people who tended not to go out often before the pandemic (i.e., 2–3 days a week, 1 day a week, or 1–3 times a month or less) tended to continue not going out often. Accordingly, in this study, we classified participants into three groups according to the changes in the frequency of going out mentioned above: the previously active group, the remained active group, and the inactive group. Overall, only 63 individuals had an increased frequency of going out during the pandemic and were excluded from this analysis, leaving data from 6,978 individuals for the final analysis.

### Statistical Analysis

Changes in the proportion of individuals reporting having a sense of *ikigai* were compared between the three groups using the chi-squared test. Changes in mental health as a change in total score between before and during the COVID-19 pandemic were compared between the groups using one-way analysis of variance. Representative values are presented as mean ± standard deviation. Data were analyzed using SPSS (Windows version 27.0, Japan IBM, Tokyo, Japan). P values < 0.05 were considered statistically significant in all analyses.

## Results

**Fig. 1:**
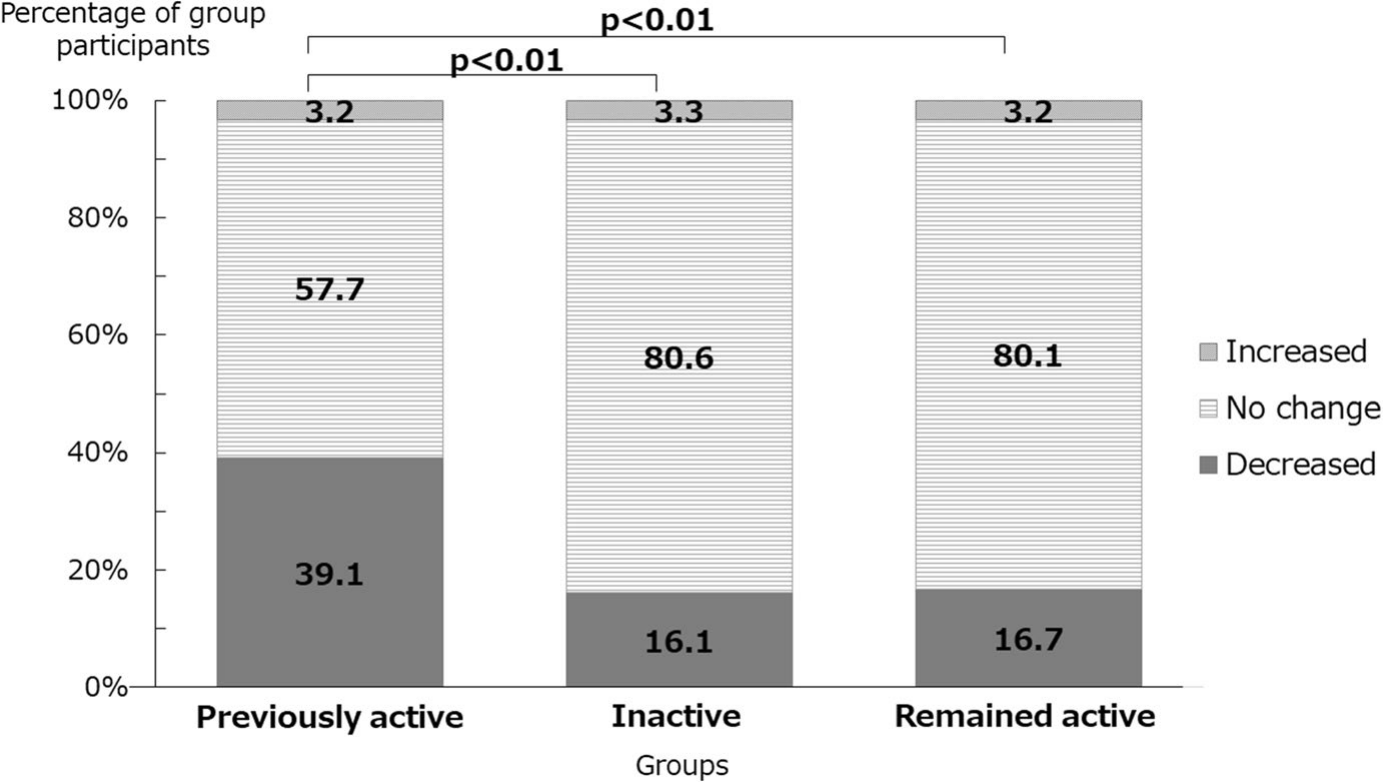
Changes in the proportion of individuals in each study group who reported having a sense of purpose in living (*ikigai*) during the COVID-19 pandemic compared with before it

Table 1 shows the demographic characteristics of the 6,978 respondents (50.6% female [n = 3,532]; mean age, 67.8 ± 12.2 years; mean body mass index, 23.0 ± 3.6 kg/m^2^). In the previously active group, *ikigai* was decreased during the pandemic in 39.1% of individuals, remained unchanged in 57.7%, and increased in 3.2%. The corresponding changes in *ikigai* were similar between the inactive and remained active groups: 16.1%, 80.6%, and 3.3% in the inactive group and 16.7%, 80.1%, and 3.2% in the remained active group, respectively. The previously active group had a significantly higher proportion of individuals whose *ikigai* decreased compared with the other groups (p < 0.05; Fig. 1).

**Table 1:** Demographic characteristics of the study participants (N = 6,978)

	Overall n = 6,978	Previously active n = 1,299	Inactive n = 2,390	Remained active n = 3,289	p-value
< 64 years old, n (%)	2,463 (35.3)	429 (33.0)	683 (28.6)	**1,351 (41.1)**	< 0.001
65–74 years old, n (%)	2,251 (32.3)	**455 (35.0)**	731 (30.6)	1,065 (32.4)	
≥ 75 years old, n (%)	2,264 (32.4)	415 (31.9)	**976 (40.8)**	873 (26.5)	
Women, n (%)	3,532 (50.6)	**749 (57.7)**	**1,298 (54.3)**	1,485 (45.2)	< 0.001
BMI ≥ 25, mean (SD)	1645 (24.1)	310 (24.4)	546 (23.4)	789 (24.5)	0.607
Current drinker, n (%)	2,558 (37.3)	389 (30.4)	*742 (31.6)*	**1,427 (44.0)**	< 0.001
Current smoker, n (%)	958 (13.9)	127 (9.9)	317 (13.5)	**514 (15.8)**	< 0.001
Living alone, n (%)	908 (13.4)	179 (14.2)	332 (14.4)	397 (12.4)	0.065
Employed, n (%)	2,564 (39.5)	338 (27.7)	*642 (29.4)*	**1,584 (51.2)**	< 0.001
University graduate education, n (%)	873 (12.9)	153 (12.1)	239 (10.3)	**481 (15.0)**	< 0.001
Experiencing financial difficulties, n (%)	1,738 (25.6)	316 (25.0)	567 (24.4)	855 (26.6)	0.151

Adjusted residuals > 1.96 = **bold**, < -1.96 = underline

**Fig. 2:**
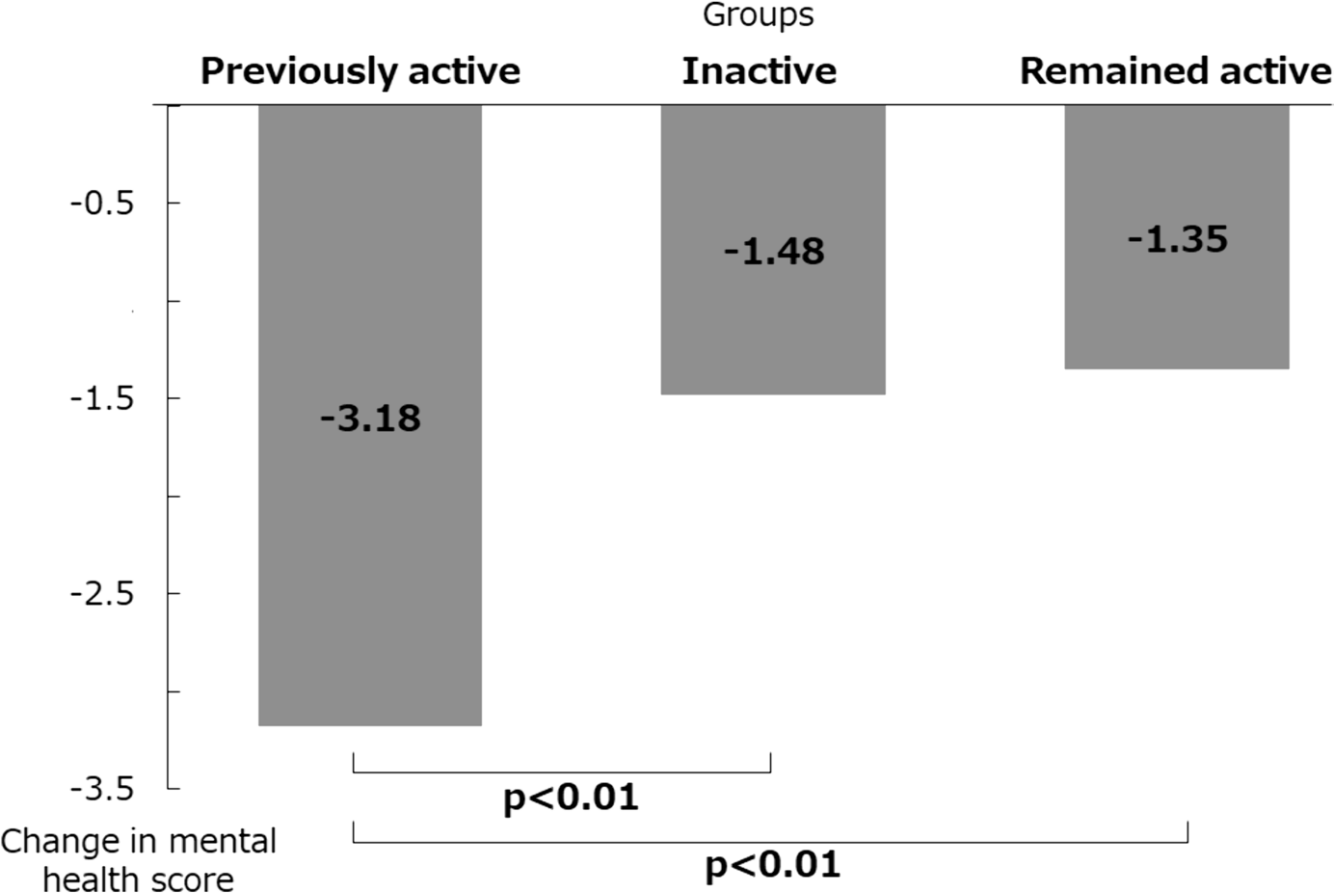
Changes in mental health scores between before and during the COVID-19 pandemic reported by the three study groups

Mental health scores analyzed with demographic characteristics as covariates decreased in all three groups during the pandemic, dropping most in the previously active group (-3.18), followed by the inactive group (-1.48) and then the remained active group (-1.35). The mental health score of the previously active group showed a significant decrease compared with the other groups (p < 0.01; Fig. 2).

## Discussion

The previously active group, who went out less often during the COVID-19 pandemic than before it, showed a significant decrease in *ikigai* and mental health compared with the inactive and remained active groups. These results are consistent with those of previous studies that found individuals with lower mental health also tended to have lower *ikigai* (Kumagai et al., 2008). In the present study, the decrease in the proportion of individuals in the previously active group who reported *ikigai* (i.e., having a sense of purpose in life) during the pandemic was 23.0 points greater than that in the inactive group and 22.4 points greater than that in the remained active group, indicating that refraining from going out during the pandemic was associated with a decrease in *ikigai* in one in four individuals who were active before the pandemic compared with those who were not.

In Japan, the promotion of *ikigai* has been a stated policy since the Ten-Year Strategy to Promote Health Care and Welfare for the Elderly was formulated in 1989 (National Institute of Population & Social Security Research, 1989). The subsequent Five-Year Direction of Policy Health Care and Welfare for the Elderly (Gold Plan 21), which was formulated in 1999, included the development of both health promotion and *ikigai* support activities, as well as prevention measures for long-term care, with the aim of fostering a vibrant elderly population (Ministry of Health, 1999). Ryff, (1989) advocates psychological well-being defined as having a sense of purpose and direction in life. This is similar to the Japanese government’s stated goal of creating an ageless society in which people of all ages can make the most of their ambitions and abilities according to their own wishes (Cabinet Office, Government of Japan, 2018). As Japan’s population continues to age, i*kigai* is likely to become an even more important concept to consider in Japan. Moreover, the results of our study suggest that those previously active people who are going out less often than before the pandemic will have worse *ikigai* and mental health than others and the government should consider measures to address this.

Decreased physical activity during the COVID-19 pandemic has been associated with adverse physical and psychological effects. For example, a 2020 online survey of 1,600 community-dwelling older adults in Japan revealed that the total time spent engaged in physical activity significantly decreased from a median (interquartile range) of 245 (90–480) min before the COVID-19 pandemic to 180 (0–420) min during the pandemic, potentially increasing the risk of disability in the near future in older adults (Yamada et al., 2020). An observational retrospective study investigating the effects of enforced lockdown during the COVID-19 pandemic on weight change, exercise, dietary habits, and anxiety/depression in 150 outpatients with obesity in northern Italy found that after 1 month, the weight of the subjects had increased significantly by an average of 1.5 kg. In addition, the adverse mental burden linked to the COVID-19 pandemic was strongly associated with the increased weight gain (Pellegrini et al., 2020). A self-administered questionnaire survey of 528 people aged 60 years or older living outside the Spanish Garcia region during the lockdown period revealed that 65.7% of the respondents were less physically active during the lockdown than before, and 25% felt some discomfort or fear when going outside (Rodríguez-González et al., 2020).

In a previous study of the psychological effects of the Great East Japan Earthquake among survivors, *ikigai* and neighborhood and family relationships were reported as psychological factors associated with significantly heightened psychological stress responses after the disaster (Sakai & Atsumi, 2020). Based on this finding, one possible measure to help people who are interacting less often with others due to the COVID-19 pandemic and whose *ikigai* might have decreased is to actively encourage them to interact more with others around them. Also, given that older people who participated in a volunteer group at least once a month were found to have a significantly lower risk of developing depression than those who did not (Tamura et al., 2021), it appears that the risk of developing depression might be higher among people engaging in less social interaction than in those who participate more in social interaction, through activities such as volunteering. Associations between poor mental health and poor social relationships have been observed in both younger and older adults (Holm-Hadulla et al., 2021; Yang et al., 2021). Moreover, social isolation was associated with depression onset in both England and Japan, suggesting that this association is not dependent on cultural background (Noguchi et al., 2021). In China, universities shut down and students had to stay at home and practice social distancing for a long time. A longitudinal survey of 66 university students involving a structured questionnaire that collected information on demographics, physical activity, negative emotions, sleep quality, and aggressiveness level in order to assess the impacts of the COVID-19 pandemic on the mental health of university students revealed a direct negative impact on general sleep quality and reduced aggressiveness. The pandemic also had an indirect impact on general negative emotions, stress, and anxiety, with sleep quality as a mediator. Moreover, physical activity was shown to directly alleviate general negative emotions, and the maximal mitigation effect was achieved when weekly physical activity was about 2500 METs. The study’s authors suggested that a possible mitigation strategy for improving mental health would involve suitable amounts of daily physical activity and getting enough sleep (Zhang et al., 2020). In the United States, a study investigating objective pandemic-related stressors, including lack of social contact, conducted a self-reported survey of more than 11.5 million adults in order to examine the mental health effects, finding that practicing social distancing on a daily basis was predictive of significantly greater mental distress, both directly and indirectly, through its effects on anxiety about becoming sick as well as concerns about finances. Although one might expect that social distancing from people outside the home would have a greater influence on people who live alone, sub-analyses based on household composition did not support this expectation. The study’s authors stated that their findings provided further evidence that the COVID-19 pandemic harmed the mental health of adults. Furthermore, week-to-week changes in the frequency of social contact within the community was suggested to lead to changes in the respondents’ mental distress (Kraut et al., 2022). Together, these findings suggest that it is important for people to stay active and open to social activities and that measures should be introduced to actively promote this alongside measures for infectious disease control.

To our knowledge, this is the first report on how *ikigai* and mental health have been affected by going out less often, reflecting reduced social and/or physical activity, due to the COVID-19 pandemic. We believe that our findings can be helpful in developing policies or approaches in the event of a future pandemic similar to the COVID-19 pandemic. However, it must be mentioned as a limitation of this study that before the COVID-19 pandemic, the conditions examined in this study could not have been predicted in advance. In addition, physical activity might have decreased due to decreased *ikigai* and mental health, which might also be a limitation of the study. Furthermore, we were not able to assess regional differences in the potential impacts of local social and activity facility closures on *ikigai* due to the lack of relevant information; therefore, this remains an issue for further study.

In conclusion, we found that people who became less active during the COVID-19 pandemic had worse *ikigai* and mental health than the other groups studied. Engaging in and sustaining physical activity, including going out, is a crucial factor for extending healthy life expectancy (World Health Organization, 2009). It has also been reported that older people with more opportunities to interact with others, including going out and talking, and more healthy behavior have better subjective health (Hosokawa et al., 2016). We believe that continuing to engage in appropriate physical and social activities, including going out, accompanied by appropriate infection control measures, even under strict infection control restrictions, can help people to maintain *ikigai* and good mental health.

## Data Availability

The data that support the findings of this study are available from Tsukuba Wellness Research, but restrictions apply to the availability of these data, which were used for the current study, and so are not publicly available.
